# Modulating Cytokine Production via Select Packaging and Secretion From Extracellular Vesicles

**DOI:** 10.3389/fimmu.2020.01040

**Published:** 2020-05-29

**Authors:** Betsy J. Barnes, Carter C. Somerville

**Affiliations:** ^1^Center for Autoimmune Musculoskeletal and Hematopoietic Diseases, The Feinstein Institutes for Medical Research, Manhasset, NY, United States; ^2^Departments of Molecular Medicine and Pediatrics, Zucker School of Medicine at Hofstra-Northwell, Hempstead, NY, United States

**Keywords:** extracellular vesicles, biogenesis, secretion, trafficking, therapeutics, intercellular, communication

## Abstract

Cytokines are soluble factors that play vital roles in systemic function due to their ability to initiate and mediate cell-to-cell communication. Another important mechanism of intercellular communication that has gained significant attention in the past 10 years is the release of extracellular vesicles (EVs). EVs are released by all cells during normal physiology, in states of resting and activation, as well as during disease. Accumulating evidence indicates that cytokines may be packaged into EVs, and the packaging of cytokines into EVs, along with their ultimate secretion, may also be regulated by cytokines. Importantly, the repertoire of biomolecules packaged into EVs is shaped by the biological state of the cell (resting vs. activated and healthy vs. disease) and the EV biogenesis pathway involved, thus providing mechanisms by which EV packaging and secretion may be modulated. Given the critical role of cytokines in driving acute and chronic inflammatory and autoimmune diseases, as well as their role in establishing the tumor immune microenvironment, in this review, we will focus on these disease settings and summarize recent progress and mechanisms by which cytokines may be packaged within and modulated by EVs, as a therapeutic option for regulating innate and adaptive immunity.

## Introduction

Intercellular communication is an essential biological feature that is mediated through (1) cell-cell contact, (2) soluble factors (cytokines, growth factors, hormones, neurotransmitters) and (3) the more recently discovered extracellular vesicles (EVs) that carry cytosolic, nuclear and cell-surface proteins, lipids, nucleotides, microRNA, and metabolites (1, 2). These three mechanisms of intercellular communication help to ensure that homeostasis is maintained in a biological system and that the system can respond appropriately to conditions of stress and disease. Conversely, dysregulation of any of these mechanisms of intercellular communication may promote altered physiology leading to disease.

Cytokines are small, non-structural proteins with low molecular weights that are synthesized and secreted by immune cells: macrophages, B and T cells, dendritic cells, neutrophils, mast cells, as well as endothelial, epithelial, fibroblasts and stromal cells, as a mechanism to communicate with each other (3). As soluble factors, they are largely responsible for promoting and regulating an immune response by acting on receptors at the cell membrane. This results in the downstream regulation of signaling molecules that stimulate cells toward sites of inflammation, infection, and trauma (4). Thus, cytokines have significant roles in a variety of functions including cell activation, differentiation, proliferation, trafficking, inflammation, and tumorigenesis that affect every organ system in the body. Their pleiotropic function(s) as intercellular messengers allows them to act at the site they are produced (autocrine), on nearby cells (paracrine), or on distant cells and tissues (endocrine), which also enables them to be self-regulating (4, 5). Cytokines act as extracellular ligands for specific membrane receptors present on responsive target cells and thus must possess a high affinity for each other. The high affinity helps to explain why cytokines can exert their biological effects in picomolar concentrations (4). As such, it is not surprising that multiple mechanisms have evolved that allow for the fine-tuning of cytokine secretion that enables an effective but limited response. This level of control is necessary in order to prevent excessive and/or dysregulated release that could drive acute and chronic inflammatory and autoimmune diseases (5, 6). As a result, it is important to understand the secretory (exocytic) pathways and endocytic compartments involved in cytokine transport, along with the regulatory molecules and cellular machinery that determine the levels and timing of cytokine release [reviewed in (5–9)]. Although cytokines are considered soluble factors, recent data indicate that they can also function as membrane proteins and be packed and stored in secretory granules, lysosome-related organelles, or secretory lysosomes and later released at the cell surface (8). Accumulating evidence indicate that cytokines can also reach the extracellular space through EVs.

EVs are a heterogeneous collection of small membrane-bound organelles that are naturally released from all cells [recently reviewed in (10)]. Originally, they were described as small vesicles that selectively remove excess and unnecessary components of cells in order to maintain homeostasis. However, subsequent studies over the past 10 years reveal that EVs play an important and targeted, functional role in cell-to-cell communication (11). Studies from multiple labs show that the packaging of cellular components within EVs are determined, in part, by the cell type they are secreted from and the physiologic status of the parental cell (12–15); the latter involving mechanisms that can be manipulated to potentially alter the cellular components within EVs and the secretion of EVs.

Based on biochemical and microscopic characterization of EVs, they can be broadly separated into two classes—exosomes and microvesicles—that are primarily distinguished by the mechanisms of biogenesis, as well as size (11). Details of the mechanisms of EV biogenesis have recently been reviewed (10). Briefly, exosomes range in size between ~50 and 150 nm in diameter (~100 nm on average) and arise from the endo-lysosomal trafficking pathway during the formation of multivesicular bodies (MVBs). Exosomes are released extracellularly when MVBs fuse with the plasma membrane. Microvesicles, on the other hand, are organelles generated by pathways that direct the outward budding or shedding of the plasma membrane and range in size between ~50 nm to 1 μm. More recent data in the field of EVs indicate that these two classes also differ by the cellular components that are packaged inside, likely resulting in different biological functions (11). The unique profile of cellular components that are packaged in EVs and secreted from a cell represents a molecular, biological, and cellular code that contains information about the parental cell at the time of secretion and how the EVs may reprogram recipient, adjacent cells and tissues during normal homeostasis and disease (14). However, precise identification of the origin of EVs is made difficult by the fact that there is substantial commonality in size, external markers, and internal content between exosomes and microvesicles. As a result, it is often not possible to definitively establish the method of biogenesis of isolated EVs, underscoring the importance of clearly defining the parameters used to identify specific EV populations (16). In this review, we provide an overview of cellular states and mechanisms by which cytokines may be packaged within and their release controlled by EVs.

## Packaging of Cytokines in EVs

While all innate immune cells have the capacity for constitutive exocytosis, their release can also occur through regulated secretory pathways [reviewed in (9, 14)]. The constitutive and induced secretion of cytokines as soluble factors provides a systemic release that helps to maintain normal homeostasis. Regulated secretion, on the other hand, provides the ability to orchestrate the rapid delivery of a concentrated amount of cytokines to a specific site in response to a specific signal (9). Recent work by Fitzgerald et al. revealed that cytokine packaging into EVs was a general biological phenomenon that occurs *in vitro, ex vivo* and *in vivo* from multiple cell types and tissues. Somewhat surprising, they found that all cytokines could be packaged into EVs. However, depending on the biological system and cell type, they reported that a cytokine could be released either in soluble or EV-associated form. Analysis across multiple biological systems (placental villous explants, tonsil explants, amnion explants, cervix explants, plasma, T cells, amniotic fluid, monocytes) revealed that 9 cytokines—Interleukin 6 (IL6), IL8, IL13, IL16, IP10, MCP1, MIP1α, MIP1β, and MIP3α–were more often found in soluble form. Conversely, 11 cytokines—IL2, IL4, IL12p70, IL17, IL21, IL22, IL33, IFNγ, ITAC, TGFβ, and TNFα–were found in greater levels in EVs. An interesting aspect of this study that is relevant to disease was the finding that cytokines packaged into EVs are not detected by standard cytokine assays, such as ELISA or other multiplexed immunoassays, since they are hidden from antibody detection by the EV membrane. Thus, methods to determine cytokine production from EVs will be important for our understanding of their role(s) in health and disease (12, 17).

What exactly is the biological meaning of packaging cytokines in EVs? Given that cytokines can exert their biological effects in picomolar concentrations, packaging cytokines into EVs is one mechanism whereby cytokine expression may be concentrated at the surface of other cells that might not otherwise be targeted by cytokines in soluble, circulating form. Further, EV packaging may facilitate cytokine delivery and targeting to distant cells. This could be mediated by binding of EV-surface cytokines to cells that express specific cytokine receptors. Another possibility is that EVs protect cytokines from environmental degradation. Indeed, Fitzgerald et al. found that EV-associated cytokines were protected from trypsin digestion, as compared to soluble cytokines (12). This protection extends to cytokines bound to the surface of EVs as well, since an 189 amino acid isoform of VEGF was found to associate with heparin on the surface of small cancer-derived EVs, resulting in reduced recognition by the VEGF antibody bevacizumab (18). Interestingly, these data suggest a mechanism by which vesicle surface-bound VEGF contributes to bevacizumab resistance in cancer patients that is likely different than soluble VEGF function. Further, synovial fibroblasts from patients with rheumatoid arthritis were shown to release EVs that express membrane-associated TNF that reduces the activation-induced cell death of CD4^+^ T cells (19). Differences in biologic function between soluble and membrane-bound cytokine receptors have been relatively well-characterized in the literature, showing that soluble receptors will often act as antagonists to membrane-bound forms (20, 21). However, comparatively little is still known regarding the different biologic functions of soluble vs. vesicle membrane-bound cytokines. As a result, the mechanism(s) by which cytokines are packaged in EVs, by internalization as vesicle cargo or expression on the vesicle surface, and how they are released from EVs, through lysis or uptake by a target cell, all contribute to the complex mechanisms of normal (healthy) and disease-related cytokine signaling.

Although the previous 5–10 years have shown rapid advancements in the field of EV research, there remain a number of unanswered questions regarding differential biological outcomes from cytokines (and other proteins) released by EVs into the microenvironment. For instance, Rana et al. reported that poly(I:C) could induce the release of both soluble and EV-secreted IL36γ from keratinocytes (22). The authors postulated that these two mechanisms of cytokine release may modulate both local and systemic immune responses to viruses and other pathogens. However, it remains unknown whether soluble and packaged IL36γ have different biological functions on target cells. Moreover, it is not currently known whether cytokine signaling in a target cell is altered dependent on how the target cell “sees” the cytokine. This lack of knowledge is partially due to the fact that multiple mechanisms exist for how target cells interact with EVs, thus adding to the complexity of our understanding of differential function [reviewed in (23)].

## Cell Type and Physiologic Status Determine Cytokine Packaging

As alluded to above, Fitzgerald et al. recently reported that medium from cultured cells and tissue explants, as well as body fluids, contained different amounts of EVs with different levels and types of cytokines. Importantly, they found that the distribution of cytokines between soluble and EV-associated forms was largely dependent on the cellular system rather than the cytokine being secreted. For example, tissue explants that contain cells in close proximity to other cells normally found in their *in vivo* microenvironments tended to release more cytokines in soluble form than were found in T cell or monocyte suspensions or in plasma. Indeed, a greater proportion of EV-associated cytokines were found from the cells and plasma. However, upon stimulation of cells, they found that the number and pattern of cytokines packaged in EVs changed depending on the stimulus, suggesting that the packaging of cytokines in EVs is not simply the property of a particular cytokine, but rather a tightly controlled biological process. For instance, stimulation of tonsillar explants with pokeweed mitogen resulted in a drastic change in the pattern of cytokine release with a shift toward more soluble secretion rather than EV-associated secretion. In contrast, human primary monocytes stimulated with either LPS or polyI:C resulted in more EVs being secreted with different patterns of cytokines associated with EVs; distinct patterns of soluble vs. EV-associated cytokine secretion were also detected between the two stimuli (6, 12).

Stimulation of human umbilical cord blood-derived mast cells by cross-linkage of FcIgE receptors (FcεRI) induces the release of granule-associated mediators such as histamine, metabolites, and cytokines (24–26). Kandere-Grzybowska et al. found that stimulation of human mast cells with IL1 rather than FcεRI cross-linking resulted in the exclusion of IL6 from secretory granules, and instead found that IL6 was secreted in 40-80 nm vesicular structures (27). Similarly, a number of reports have been recently published showing that exosomes from the plasma of HIV-infected individuals have distinct levels and types of cytokines as compared to exosomes from healthy donors (28–30). Interestingly, in patients with diabetes, the association of specific cytokines with EVs was found to be strongly influenced by disease duration and treatment outcome (31). Altogether, these data support that EV-associated cytokine loading and secretion may be directed in a cell type- and stimuli-dependent manner.

## Biological Activity of EV-Associated Cytokines

In the mid-1990s, EVs secreted from B cells were shown to have an immunological function in antigen presentation and as vesicles that can induce T cell responses (32–34). We now know that one of the mechanisms by which EVs elicit immunological function is that they can serve as alternate carriers for the delivery of cytokines. Immunologically, EVs maintain characteristics of the antigen presenting cell (APC) that they were derived from, exposing the extracellular domain of major histocompatibility complex (MHC) molecules at the vesicle surface. Thus, EVs released by APCs carrying surface MHC Class I and MHC Class II can directly stimulate CD8^+^ and CD4^+^ T cells, respectively [reviewed in (33)]. Of note, EVs are also generated from immunosuppressive APCs. For instance, autologous EVs isolated from plasma shortly after antigen (Ag) stimulation could be used to induce Ag-specific immunosuppression (35, 36). Further, EVs isolated from bronchoalveolar lavage fluid following Ag-specific exposure could be used to prevent Ag-specific allergic responses (37, 38). Last, EVs present in human breast milk and colostrum were found to increase the number of T regulatory (T_reg)_ cells and thus could be used to suppress immune responses (37). In this context, pregnancy has been shown to alleviate the severity of some autoimmune diseases, such as rheumatoid (RA) arthritis and multiple sclerosis (MS) (39).

Given the small size of EVs, they are capable of crossing major biological barriers such as the blood-brain barrier, and thus provide interesting prospects for therapeutic packaging and regulation (40–42). It is now well-recognized that EVs have a wide range of pleiotropic functions in multiple biological processes. For example, in an *in vitro* model of cardiovascular disease, EVs isolated from TNFα-induced human vascular endothelial cells (HUVEC) were taken up by monocytes and un-induced HUVEC, promoting an inflammatory response (13, 43, 44). Hosseinkhani et al. reported a select increase in IL6, IL8, and ICAM1 levels in un-induced HUVEC after co-incubation with EVs isolated from TNFα-induced HUVEC, while THP1 cells showed an increase in ICAM1, MIP1β, CCL5, and CXCL10 levels (13). The change in THP1 inflammatory mediators by EVs led to an increase in monocyte adhesion and migratory function. Another interesting study reported that exosomes isolated from mesenchymal stem cells (MSCs) of human umbilical cord treated with interferon (IFN)γ or a combination of TGFβ plus IFNγ contained increased levels of TGFβ, IDO, IL10, and IFNγ that, when incubated with PBMCs, resulted in increased numbers of T_regs_ (45).

In HIV-positive individuals, cytokines were found to be markedly enriched in exosomes and exposure of these exosomes to purified naïve peripheral blood mononuclear cells (PBMCs) resulted in the induction of CD38 expression on naïve and central memory CD4^+^ and CD8^+^ T cells, likely contributing to viral propagation via activation of bystander cells (30). An independent study characterizing plasma EVs from HIV-positive individuals found increased oxidative stress markers that correlated with an IFN gene signature and immune activation (28). Another interesting immunologic function for EVs was discovered in the placenta as a mechanism to regulate immunity against the fetus during pregnancy. Holder et al. reported that macrophage-derived exosomes containing IL6 and IL8 were actively transported into the human placenta to stimulate placental cytokines (46).

## EV-Associated Cytokines in Autoimmune Disease

Recent evidence supports that EVs can mediate immune stimulation or suppression and can drive inflammatory, autoimmune and infectious disease pathology (47–49). One of the mechanisms by which EVs can drive autoimmune disease is that they serve as carriers of pathogen-associated and damage-associated molecular patterns (PAMPs and DAMPs, respectively), as well as cytokines, autoantigens and tissue-degrading enzymes (48). Indeed, synovial EVs from patients with RA were found to contain citrullinated proteins and, in the autoimmune disease systemic lupus erythematosus (SLE), EVs could serve as autoantigens in the formation of immune complexes (50–54). In addition, cytokines, such as IL6, are highly implicated in the development and progression of multiple autoimmune diseases whose production can be regulated by EV packaging and secretion. The role of IL6 in autoimmune disease pathogenesis is due in part to its influence on CD4^+^ T cell lineage and regulation [reviewed in (55)]. We provide examples in the above sections of how IL6 packaging and secretion in EVs can be regulated by different stimuli.

**Figure 1:**
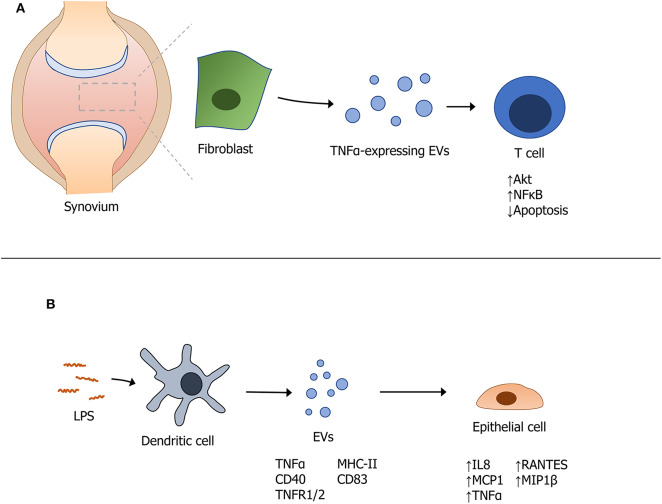
Modulation of autoimmunity by extracellular vesicles expressing TNFα. **(A)** Fibroblast-derived EVs containing TNFα modulate T cell function in the synovium of patients with rheumatoid arthritis. **(B)** Stimulation of dendritic cells with LPS induces the packaging and secretion of specific EV-associated cytokines that themselves, induce a downstream effect(s) on cytokine production from epithelial cells.

Another cytokine that contributes to autoimmune disease pathogenesis is TNFα. High levels of circulating TNFα are a major driver of RA. Interestingly, a membrane-bound form of TNFα was recently detected from individuals with osteoarthritis (19). The premise that EVs package cytokines that contribute to the amplification of an immune response was supported by work from Obregon et al. revealing the presence of large amounts of TNFα packaged into EVs derived from LPS-activated dendritic cells (DCs). These EVs also contained MHC II, CD40, CD83, TNFR1, and TNFR2 and were internalized by epithelial cells that became activated to release cytokines and chemokines such as IL8, MCP1, MIP1β, RANTES, and TNFα (56). In another related study, Zhang et al. identified a membrane bound form of TNFα on exosomes produced from synovial fibroblasts of patients with RA. These exosomes were found to activate Akt and NFκB pathways and rendered T cells resistant to undergo apoptosis; the authors proposed that this contributed to T cell-mediated pathology in RA (19). Figure 1 provides an overview of these mechanisms through which EVs expressing TNFα modulate autoimmunity.

IL1β is a pro-inflammatory cytokine that has stimulatory effects and helps promote the differentiation of CD4^+^ T cells into T helper 1 (Th1) and Th17 lineages, both of which are known to contribute to autoimmune disease pathogenesis (57, 58). The release of the active form of IL1β follows a finely regulated process [(59); reviewed in (60)] and we now know that EV production plays a role in the maturation process of IL1β (61–66). This is dependent on the formation of the inflammasome, a multiprotein complex of innate immunity that is also involved in the secretion and loading of proteins associated with vesicles (67, 68). Different types of stimuli have been reported to promote inflammasome activation resulting in IL1β secretion via EVs, such as extracellular ATP that serves as a strong activator of the NRLP3 inflammasome, resulting in increased release of EVs (65, 69). Another stimulus is ionic fluxes that cause membrane polarization. It has been well-established that Ca^2+^ influx causes inflammasome activation and vesicular production. Ca^2+^ influx also induces the activation of different calcium-dependent proteins involved in membrane and cytoskeletal modification, thus facilitating the release of EVs (70). Last, a non-canonical route for inflammasome activation and the maturation of IL1β involves caspase 4/5, which directly recognize intracellular LPS. Caspase 4/5-mediated activation of the inflammasome strongly induces the release of IL1β, IL18, and other EV-associated cytokines (63).

High serum levels of type I (IFNα), II (IFNγ), and III (IFNλ1) are observed in patients with SLE and have been associated with high disease activity; thus, IFNs are considered to be key molecules in the pathogenesis of SLE (71–74). Interestingly, before EVs were identified as entities with physiologic function, it was well-known that IFNs were able to affect enveloped virus budding, release, and infectivity by increasing the expression of genes encoding restriction factors, such as ISG15 that has regulatory functions in EV packaging and secretion (75). ISG15 is an IFN stimulated gene (ISG) and an ubiquitin-like modifier (76–78). It has been identified in microvesicles and exosomes originating from TLR3 (polyI:C)-activated human brain microvascular endothelial cells (79). Importantly, ISG15 was found to ISGylate TSG101, which is a component of the ESCRT-I complex that mediates ESCRT-dependent EV secretion [reviewed in (10, 14, 80)]. Thus, not surprisingly, ISGylation was reported to influence exosome secretion. Villarroya-Beltri et al. revealed that type I IFNs trigger TSG101 modification via ISG15 that results in TSG101 degradation and impaired exosome secretion (81, 82). They reported that ISGylation of TSG101 triggers MVB co-localization with lysosomes, thus promoting the aggregation and degradation of MVB proteins, and the ultimate impairment of exosome secretion (81). Relevant to type I and II IFNs, the transcription factor interferon regulatory factor 1 (IRF1) was found to regulate select GTPases, such as Rab27a that is a key factor in EV secretion. Yang et al. found that IFNγ-induced IRF1 upregulation promoted Rab27a expression and EV secretion; conversely, knockdown of IRF1 or Rab27a resulted in reduced EV secretion (83). In addition to contributing to the regulation of EV secretion, IFNs also contribute to the packaging of its cellular components [reviewed in (6)].

## EV-Associated Cytokines in Cancer

In cancer, tumor-derived EVs have been shown to play roles in immune evasion and metastatic progression (84–87). One of the first studies revealed that vaccination of mice with exosomes isolated from tumor peptide-pulsed DCs primed tumor-specific cytotoxic T cells and suppressed tumor growth in a T cell-dependent manner (88). Similarly, Seo et al. found that EVs released from activated CD8^+^ T cells of healthy mice were capable of attenuating tumor invasion and metastasis by apoptotic depletion of mesenchymal tumor stromal cells (89). Subsequent studies of EVs secreted from melanoma and prostate cancer cells revealed that they express programmed death-ligand 1 (PD-L1) on their surface, which suppresses the function of CD8^+^ T cells and facilitates tumor growth (90–93). The level of PD-L1 expression was found to correlate with disease stage, and was increased by IFNγ stimulation (94). Importantly, the associated suppression of CD8^+^ T cell response by exosomal PD-L1 could be abrogated by treatment with PD-1 or PD-L1 inhibitors to induce immune-mediated reduction of tumor growth.

Most solid tumors exhibit increased release of EVs, accompanied by alterations in their composition of proteins, lipids, and genetic material (95, 96). As a result, tumor-derived EVs have diverse effects on tumor growth, invasion, metastasis, and immune response, in part, through their modulation of cytokine production by cells of the innate and adaptive immune system (86). The complex interplay between the diverse array of cells in the tumor microenvironment and the pleiotropic factors that are secreted, is the subject of extensive current research, and our knowledge of exactly how these cells and mediators interact is incomplete. Nonetheless, it is clear that EVs promote tumor growth and progression in most solid tumors, highlighting the importance of these mediators in tumor-immune regulation.

Tumor-associated macrophages (TAMs) are major regulators of inflammation and immune response in the tumor microenvironment and are thus important targets of tumor-derived EVs. Crosstalk between tumor-derived EVs and macrophages can polarize them toward a more M2-like, pro-tumor TAM (97), which is associated with higher levels of the immunosuppressive cytokines IL10, IL4, and TGFβ. However, EVs can also promote tumor progression through an increase in pro-inflammatory functions of macrophages. Wu et al. found that exosomes secreted by gastric tumors were capable of inducing pro-inflammatory signaling in macrophages via activation of NF-κB, thereby promoting tumor growth and invasion (98). Similarly, breast cancer exosomes were found to induce macrophage-mediated secretion of the cytokines TNFα, IL6, and MCP1, which stimulate tumor progression and metastasis (99, 100). Increased IL6 production mediated by tumor-derived exosomes results in suppressed dendritic cell activity and attenuated immune response, resulting in enhanced tumor growth (101).

Tumor-derived exosomes also promote tumor growth through the stimulation of myeloid-derived suppressor cells (MDSCs), which have immunosuppressive effects in tumors. Multiple cancer types have been found to secrete exosomes containing heat shock proteins, Hsp72 and Hsp90, which activate Stat3 in MDSCs via IL6 and promote immunosuppression and tumor growth (85). Exosomes isolated from B16 melanoma tumors in mice were shown to stimulate MDSCs to produce TNFα, MCP1, and IL6 in a MyD88-dependent manner, which promotes immunosuppression, tumor growth, and metastasis (23). Not surprisingly, these same pro-inflammatory cytokines were implicated in ovarian cancer, in which exosomes isolated from the body fluids of patients induced production of IL1β, TNFα, and IL6 by THP-1 monocytes (102).

**Figure 2:**
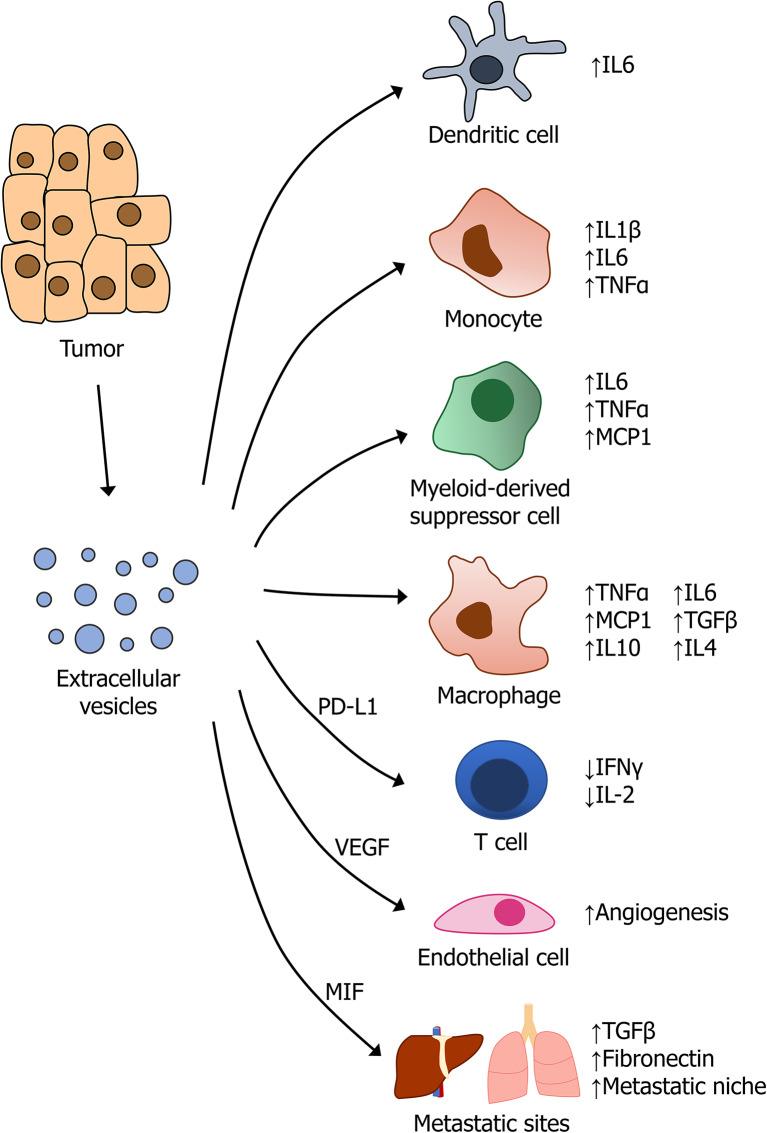
Regulation of cytokine signaling by tumor-derived extracellular vesicles.

Last, tumor-derived EVs have been implicated in the development of pre-metastatic niche (PMN) formation in a variety of cancers [recently reviewed in (103)]. Results from pancreatic cancer, breast cancer, ovarian cancer, and melanoma, among others, highlights the importance of EVs in regulating intercellular communication at sites distant from the primary tumor (104). For example, in a well-characterized model of pancreatic cancer, tumors were found to secrete exosomes containing macrophage migration inhibitory factor (MIF), which induces TGFβ signaling in Kupffer cells in the liver. This resulted in increased production of fibronectin by hepatic stellate cells, creating an environment that is more permissive to metastatic colonization by tumor cells (105). In response to the hypoxic microenvironment that is present in most solid tumors, many types of cancers were found to promote endothelial growth through the release of pro-angiogenic factors, such as VEGF, that can also be packaged inside exosome (18, 106, 107). A summary of EV-mediated regulation of cytokine production by cells in the tumor microenvironment is provided in Figure 2.

## Modulating EV Secretion as a Mechanism to Control Cytokine Release

Although EVs are released in resting cells, stimulating events, such as cell activation, leads to increased intracellular calcium levels, resulting in cellular membrane remodeling and enhanced EV secretion (108). Pharmacologic modulation of EV output can be achieved through treatment with agents that interfere with cytoskeletal remodeling that is required for the formation of MVBs and trafficking of proteins into vesicles for their subsequent release (10, 80). Calpains are a family of calcium-dependent cysteine proteases that are important for unconventional protein secretion and inflammasome activation (65). Inhibition of calpain with a small-molecule inhibitor, such as MDL28170, blocks vesicular formation and the subsequent release of EVs (65). Given the role of caspase 4/5 in inflammasome activation and release of IL1β-associated EVs, the use of a caspase 4 inhibitor was found to block EV secretion from LPS-stimulated human macrophages (63). Treatment of cells/tissues with the microbial metabolite Manumycin A, a farnesyltransferase inhibitor, resulted in decreased EV biogenesis and secretion via modulation of ESCRT machinery (12, 109). A similar pharmacologic approach is to inhibit the formation of MVBs by inhibiting sphingomyelinase activity. Sphingomyelinases are required for the inward budding and eventual release of MVBs through an ESCRT-independent pathway. GW4869 is a neutral sphingomyelinase inhibitor that inhibits vesicle formation (110). Last, simvastatin was recently identified as an inhibitor of EV secretion based on the rationale that cholesterol is necessary for the formation of vesicle membranes. However, simvastatin's function as an HMG-CoA reductase inhibitor does not entirely explain the mechanism, as supplementation with mevalonate did not fully restore EV output to baseline levels (111). Given that different mechanisms of EV biogenesis exist, we may utilize this knowledge to selectively target (inhibit) specific populations of EVs while leaving other subsets of EVs untouched.

As our current understanding of the mechanisms that differentially regulate the packaging of cytokines in EVs from resting and activated cells expands, this knowledge may be also used to preferentially drive the packaging of distinct groups of cytokines into EVs for therapeutic use. For instance, DCs can be stimulated to secrete EVs that induce the differentiation of immunosuppressive T_regs_ for the treatment of autoimmune disease (112–116). The regulation of T cell differentiation to immunosuppressive states is already under consideration for the treatment of autoimmune disease (49, 117, 118). Last, determination of the molecular machinery required for EV-associated cytokine secretion, such as ESCRT-dependent or -independent and autophagy-dependent, will provide critical information on select treatments that may target specific pathways [recently reviewed in (119)].

## Engineering EVs to Therapeutically Deliver Cytokines

EV encapsulation of cytokines may facilitate their delivery and targeting to distant cells (34). Recent work has demonstrated the feasibility of engineering EVs to take up proteins as cargo (120–122), presenting a number of techniques by which EVs could be artificially generated to carry cytokine payloads to distant sites. An advantage to this method is that it does not require *a priori* knowledge of the biogenesis pathway resulting in EV cargo loading and secretion. Alternatively, EVs may be targeted to specific cells via binding of EV surface cytokines to cells that express the specific cytokine receptor (123). Sialic-acid binding immunoglobulin lectins, C-type lectins, lactadherin, MHC I, and II receptors, transferrin receptors, tetraspanins, and viral proteins have all been identified as molecules that may promote EV targeting (124–129). Thus, enrichment of exosomes on the basis of their surface ligand expression or ligand enrichment on engineered EVs may be used to induce or inhibit signaling events in recipient cells or to develop receptor-mediated tissue (and cell) targeting (80). Here, we provide two examples of how EVs can be therapeutically modulated for packaging of specific cytokines that drive an immune response. The first example is treatment of bone marrow-derived mast cells with IL4 to drive secretion of exosomes that express MHC II, CD86, LFA-1, and ICAM1, resulting in activation of the adaptive immune arm by inducing proliferation of B and T cells *in vitro* and *in vivo* (130). The second example is from engineering tumor cells to overexpress CD40L, resulting in tumor-derived exosomes that overexpress CD40L to promote dendritic cell maturation, resulting in increased T cell proliferation and antitumor activity *in vivo* (131).

While technological advances in isolating, characterizing, and now engineering EVs to deliver therapeutic payloads and immune modulators are being made [recently reviewed in (80)], it is not until the biological mechanisms by which cytokines are selectively packaged into EVs and the molecular machinery required for secretion determined, that we will be able to fully harness the potential of this natural, physiologic mechanism for cytokine modulation in the context of disease.

## Author Contributions

BB and CS planned and prepared the manuscript.

## Conflict of Interest

The authors declare that the research was conducted in the absence of any commercial or financial relationships that could be construed as a potential conflict of interest.
